# Pre‐Emptive TIPS for Acute Variceal Bleeding: A Systematic Review and Meta‐Analysis of Randomized Controlled Trials

**DOI:** 10.1155/ijh/3244544

**Published:** 2026-09-02

**Authors:** Wan Muhammad Izhar bin Wan Ismail, Susmitha Chalamalasetti, Faraz Iqbal, Karan Garg, Aashni Purohit, Hariniska Jayaraman Kannan, Kalyanramnaidu Botsa, Basel Al Khatib, Yaseen Sulaiman, Amar Abdalrazak, Muhammad Safiullah, Asma’a Munasar Ali Alsubari, Adnan Bhat, Muhammad Abu Zar, Prasun K. Jalal

**Affiliations:** ^1^ Department of Gastroenterology, London North West University Healthcare NHS Trust, London, UK; ^2^ Department of Internal Medicine, St. Francis Medical Center, Monroe, Louisiana, USA; ^3^ Department of Medicine, Hull University Teaching Hospitals, Hull, UK, duhs.edu.pk; ^4^ Department of Medicine, Surat Municipal Institute of Medical Education and Research, Surat, India, smimer.suratmunicipal.gov.in; ^5^ Department of Medicine, KAP Viswanatham Government Medical College, Tiruchirappalli, India, duhs.edu.pk; ^6^ Department of Medicine, Sri Ramachandra Institute of Higher Education and Research, Chennai, India, sriramachandra.edu.in; ^7^ College of Medicine, Alfaisal University, Riyadh, Saudi Arabia, alfaisal.edu; ^8^ Department of Surgery, Jordan University Hospital, Amman, Jordan, ju.edu.jo; ^9^ Department of Medicine, King Edward Medical University, Lahore, Pakistan, kemu.edu.pk; ^10^ Faculty of Medicine, Sana’a University, Sana, Yemen, su.edu.ye; ^11^ Division of Hospital Medicine, University of Florida, Gainesville, Florida, USA, ufl.edu; ^12^ Department of Internal Medicine, UT Southwestern, Dallas, Texas, USA, utsouthwestern.edu; ^13^ Section of Gastroenterology and Hepatology, Department of Medicine, Baylor College of Medicine, Houston, Texas, USA, bcm.edu

**Keywords:** acute variceal bleeding, liver cirrhosis, meta-analysis, portal hypertension, pre-emptive TIPS, randomized controlled trials

## Abstract

**Background:**

There is evolving evidence regarding whether pre‐emptive transjugular intrahepatic portosystemic shunt (p‐TIPS) improves outcomes in patients with acute variceal bleeding. This meta‐analysis is aimed at evaluating the effectiveness of p‐TIPS compared with standard care in reducing mortality and rebleeding in high‐risk patients with cirrhosis.

**Methods:**

We systematically searched CENTRAL, MEDLINE, Embase, and ClinicalTrials.gov to identify randomized controlled trials (RCTs) comparing p‐TIPS placed within 72 h of admission with standard care. All statistical analyses were performed using RevMan Version 5.4.

**Results:**

Six RCTs (*n* = 424 participants) met inclusion criteria. p‐TIPS significantly reduced all‐cause mortality (RR 0.54, 95% CI 0.39–0.76; ARR 14.9%; NNT ≈ 7) and rebleeding (RR 0.29, 95% CI 0.16–0.55; ARR 28.4%; NNT ≈ 4) compared with standard care, with consistent effects across variceal subtypes and degrees of hepatic decompensation. p‐TIPS also significantly reduced new or worsening ascites (RR 0.51, 95% CI 0.35–0.74), hepatorenal syndrome (RR 0.37, 95% CI 0.15–0.92), liver‐related mortality (RR 0.56, 95% CI 0.34–0.94), and serious adverse events (RR 0.72, 95% CI 0.59–0.88), without increasing hepatic encephalopathy (RR 1.01, 95% CI 0.72–1.43).

**Conclusions:**

p‐TIPS significantly reduced mortality and rebleeding risk in high‐risk patients with acute variceal bleeding, without increasing encephalopathy risk. These findings support its broader integration into clinical practice guidelines. Further large‐scale RCTs with extended follow‐up are warranted to define optimal patient selection, particularly in gastric variceal subgroups. PROSPERO: CRD420261329675

## 1. Introduction

Acute variceal bleeding (AVB) remains one of the most life‐threatening complications of portal hypertension in patients with liver cirrhosis. Despite improvements in standard treatment, AVB is associated with mortality rates of 15%–20%. [1, 2] Recommended standard management involves a combination of vasoactive drugs, prophylactic antibiotics, and endoscopic band ligation, with pre‐emptive transjugular intrahepatic portosystemic shunt (p‐TIPS) indicated in high‐risk patients. [3–5] Recent studies highlight considerable variation in outcomes across patient subgroups. Although the mortality rate among cirrhotic patients with esophagogastric variceal bleeding is reported to be 11.6%, this rises markedly to 21% in those with concurrent myocardial injury. [1] Evidence suggests that mortality disproportionately affects patients with advanced liver disease and moderate‐to‐severe esophageal varices, in whom standard treatment frequently fails to achieve adequate hemostasis. [1] Current international guidelines recommend early risk assessment and timely escalation of therapy, based on liver function and portal hypertension severity, to minimize the risk of complications, including variceal bleeding. [4, 6]

Transjugular intrahepatic portosystemic shunt (TIPS) achieves mechanical decompression of the portal venous circulation by creating an intrahepatic low‐resistance channel, thereby rapidly and sustainably reducing portal pressure. Historically, TIPS has been reserved as a salvage intervention for patients with refractory or recurrent variceal hemorrhage unresponsive to first‐line endoscopic and pharmacological therapy. Over the past decade, however, accumulating evidence has prompted a fundamental reconceptualization of its role in the acute setting. p‐TIPS, defined as shunt insertion within 72 h of index hemorrhage before rebleeding occurs, has emerged as a potentially transformative strategy in carefully selected high‐risk patients. [7] Previous randomized controlled trials (RCTs) have shown that p‐TIPS significantly reduces rebleeding and improves survival, particularly in patients with Child–Pugh C cirrhosis (up to 13 points) or Child–Pugh B cirrhosis with active bleeding at endoscopy. [7, 8] Real‐world studies have also reported similar effects, suggesting that early TIPS may offer consistent advantages outside of controlled trial environments. [9]

However, results across studies have varied. Existing systematic reviews have attempted to summarize the evidence, but most have combined RCTs and observational studies, making their conclusions vulnerable to confounding and selection bias. [10, 11] Others grouped different TIPS strategies together (i.e., early, elective, or rescue), limiting their relevance to acute bleeding episodes. [12] Importantly, several earlier reviews did not include the most recent GAVAPROSEC trial on pre‐emptive TIPS, which provides contemporary evidence with a larger sample size than some previous studies, a high‐quality trial design focused on gastric varices, and the potential to consolidate our clinical conclusions. [13]

To address these gaps, we conducted an updated and rigorous meta‐analysis restricted exclusively to RCTs evaluating pre‐emptive TIPS in high‐risk AVB. By excluding observational studies, incorporating the latest high‐quality RCTs, and focusing strictly on pre‐emptive intervention, this review provides the most recent assessment of the efficacy and safety of pre‐emptive TIPS.

## 2. Methods

This systematic review and meta‐analysis was conducted in accordance with the recommendations of the Cochrane Handbook for Systematic Reviews of Interventions and reported in accordance with the Preferred Reporting Items for Systematic Reviews and Meta‐Analyses (PRISMA) guidelines. [14, 15] The protocol was registered.

### 2.1. Search Strategy and Literature Search

We searched MEDLINE (via Ovid), Embase (via Ovid), the Cochrane Central Register of Controlled Trials (CENTRAL), and ClinicalTrials.gov from inception to January 2026. The search strategy combined keywords and controlled vocabulary relating to AVB, portal hypertension, and pre‐emptive TIPS. Reference lists of relevant studies were also inspected for any eligible trials.

### 2.2. Inclusion and Exclusion Criteria

Studies were selected with inclusion criteria as follows: 1) Population: Adults (≥ 18 years) with AVB secondary to cirrhosis; 2) Intervention: Pre‐emptive TIPS (TIPS placement within 72 h of hospital admission); 3) Comparison: Placebo or standard therapy, including vasoactive medication, endoscopic band ligation, and antibiotic prophylaxis; and 4) Study design: RCTs only.

The exclusion criteria were: (1) nonrandomized study designs, including observational studies, quasi‐randomized trials, case–control studies and case series; (2) studies performed in pediatric or animal populations; (3) trials evaluating elective or rescue TIPS rather than pre‐emptive TIPS or studies where outcomes for pre‐emptive TIPS could not be separated from other TIPS strategies; and (4) duplicate publications from the same trial cohort, in which case the most complete dataset was retained. No restrictions were applied regarding publication date or language.

### 2.3. Study Screening and Data Extraction

All articles retrieved from literature search were imported into Rayyan (https://www.rayyan.ai/) and duplicate reports were removed. This was followed by initial screening of titles and abstracts by two independent reviewers. Subsequently, full text review of the remaining potentially eligible articles was done. At each level, disagreements were resolved through discussion, if agreement could not be reached, a senior author was consulted.

Three independent reviewers extracted data from the included studies on a predefined Excel sheet. Extracted data included study characteristics (authors, year, country, and design), patient demographics, clinical features (Child–Pugh class, variceal types, baseline MELD score, and follow‐up time), and outcomes.

### 2.4. Outcomes

The primary outcomes were: (1) all‐cause mortality; and (2) rebleeding following pre‐emptive TIPS. Secondary outcomes included new or worsening ascites, hepatic encephalopathy, spontaneous bacterial peritonitis, hepatorenal syndrome, liver‐related mortality, and serious adverse events as defined by the individual trials.

### 2.5. Risk of Bias Assessment

Risk of bias was assessed by two independent reviewers using Cochrane RoB 2 tool [16]. Studies were rated as having a low risk of bias, high risk of bias, or some concerns. RoBvis tool was used to generate a visual summary of the quality assessment.

### 2.6. Statistical Analysis

All statistical analyses were performed on Review Manager (RevMan, Version 5.4; The Cochrane Collaboration, Copenhagen, Denmark). Outcomes were pooled using a random‐effects model and pooled estimates were presented as risk ratios (RR) with 95% CIs. Heterogeneity across studies was evaluated using Higgins *I* ^2^ statistics. Subgroup analyses were performed for the primary outcomes based on: 1) non‐GOV1 gastric variceal bleeding versus other variceal bleeding; and 2) Child–Pugh A < 25% versus > 25% in the study population.

Publication bias could not be assessed because the recommended threshold of 10 studies per outcome was not met.

## 3. Result

### 3.1. Study Selection and Characteristics of Included Studies

A total of 4218 records were imported through literature search. After removal of 477 duplicates, remaining 3741 records were screened based on title and abstract, resulting in the exclusion of 3633 records. Full texts of the remaining 108 articles were assessed for eligibility. Consequently, six studies [7, 8, 13, 17–19] were included in the final review. The complete study selection process is illustrated in the PRISMA flow diagram (Figure S1).

Six RCTs comprising a total of 424 patients with acute esophageal or gastric variceal bleeding were included. Of these, 229 patients received pre‐emptive TIPS, and 195 were assigned to standard medical or endoscopic therapy. Baseline liver disease severity varied across studies, with Child–Pugh classifications ranging from mild to moderate dysfunction and MELD scores typically between 14 and 17. Follow‐up periods ranged from 1 to 2 years. A detailed summary of study characteristics is given in Table 1.

**Table 1:** Baseline Characteristics of Included Randomized Controlled Trials.

Study	Arm	*n*	Population	Age, years	Male, %	Child–Pugh A/B/C, %	Child–Pugh score	MELD score	Follow‐up
Monescillo 2004	p‐TIPS	26	Endoscopic proven variceal bleeding, esophageal or gastric varices	56 ± 12	85	12/42/46	9.2 ± 2.0	NR	1 year
	Control	26		59 ± 11	73	15/38/46	9.2 ± 2.3	NR	
García‐Pagán 2010	p‐TIPS	32	Esophageal varices, Child–Pugh score < 13	52 ± 10	65.6	0/50/50	9.3 ± 1.8	15.5 ± 5	1 year
	Control	31		49 ± 6	74.2	0/52/48	9.5 ± 1.8	16.9 ± 6.3	
Lv 2019	p‐TIPS	86	Endoscopic proven acute variceal bleeding	50 · 7 ± 11 · 6	63	57/20/23	8 ± 1	14 ± 4.3	2 years
	Control	46		50 · 9 ± 10 · 4	76	56/22/22	8 ± 1	13.4 ± 4.6	
Dunne 2020	p‐TIPS	29	Acute esophageal variceal bleeding, Child–Pugh score < 8 and > 13	53 ± 10	91	0/13/16	9.8 ± 1.2	17 ± 3.4	1 year
	Control	29		48 ± 12	90	0/12/17	9.8 ± 1.5	17 ± 3.8	
Escorsell 2023	p‐TIPS	11	IGV1 and/or GOV2 gastric varices, Child–Pugh score 6–10	59 ± 11	72.7	36/64/0	7.3 ± 1.9	NR	1 year
	Control	10		64 ± 7	80	40/60/0	7.7 ± 1.1	NR	
Cervoni 2025	p‐TIPS	49	Non‐GOV1 gastric varices, Child–Pugh score < 13	57.7 ± 10.7	85	23/49/28	8.2 ± 2.1	14.1 ± 5.4	1 year
	Control	56		58.6 ± 8.8	76	11/58/30	8.6 ± 1.9	14.5 ± 4.7	

Abbreviations: GOV, gastro‐esophageal varices; IGV, isolated gastric varices; MELD, Model for End‐stage Liver Disease; p‐TIPS, pre‐emptive transjugular intrahepatic portosystemic shunt; NR, not reported. Data are presented as mean ± standard deviation (SD) unless otherwise stated.

### 3.2. Risk of Bias Assessment

The included trials demonstrated predominantly low risk of bias. Cervoni (2025), Dunne (2020), Garcia‐Pagán (2010), and Lv (2019) were assessed as low risk across all domains, with adequate randomization, protocol adherence, and complete outcome reporting. Escorsell (2023) raised concerns about the randomization process, whereas Monescillo (2004) exhibited a higher risk, particularly regarding deviations from intended interventions and selective outcome reporting (Figure S2).

### 3.3. Primary Outcomes

#### 3.3.1. All‐Cause Mortality

**Figure 1:**
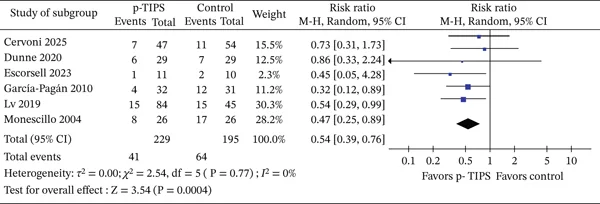
Forest plot of all‐cause mortality comparing pre‐emptive TIPS versus standard care.

Across six RCTs (*n* = 424), p‐TIPS significantly reduced all‐cause mortality compared with standard care (RR 0.54, 95% CI 0.39–0.76; ARR 14.9%; NNT ≈ 7) (Figure 1). The mortality reduction with p‐TIPS remained consistent regardless of variceal bleeding subtype/location (*p* = 0.52) or degree of hepatic decompensation (*p* = 0.93) (Figures S3 and S4).

#### 3.3.2. Rebleeding

**Figure 2:**
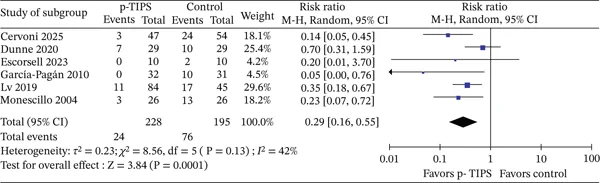
Forest plot of rebleeding comparing pre‐emptive TIPS versus standard care.

Across six RCTs (*n* = 423), p‐TIPS markedly reduced the risk of rebleeding compared with standard care (RR 0.29, 95% CI 0.16–0.55; ARR 28.4%; NNT ≈ 4) (Figure 2). The effect of p‐TIPS in reducing rebleeding was not affected by variceal bleeding type (*p* = 0.19) or liver disease severity (*p* = 0.62) (Figures S5 and S6).

#### 3.3.3. Secondary Outcomes

Pre‐emptive TIPS significantly reduced the risk of new or worsening ascites (RR 0.51, 95% CI 0.35–0.74), hepatorenal syndrome (RR 0.37, 95% CI 0.15–0.92), liver‐related mortality (RR 0.56, 95% CI 0.34–0.94), spontaneous bacterial peritonitis (RR 0.25, 95% CI 0.08–0.81), and serious adverse events (RR 0.72, 95% CI 0.59–0.88) (Figures S7, S8, S9, S10, and S11). No significant difference was observed between groups in the incidence of hepatic encephalopathy (RR 1.01, 95% CI 0.72–1.43) (Figure S12).

## 4. Discussion

This study‐level meta‐analysis of six RCTs, enrolling 424 patients with AVB secondary to cirrhosis, demonstrated that p‐TIPS significantly reduced all‐cause mortality and the risk of rebleeding compared with standard care. The mortality and rebleeding reductions were consistent across variceal subtype/location and Child–Pugh strata, and p‐TIPS additionally reduced the risk of hepatorenal syndrome, new or worsening ascites, liver‐related mortality, and serious adverse events without a significant increase in hepatic encephalopathy.

Current international guidelines recognize p‐TIPS as a recommended strategy in selected high‐risk patients with AVB, particularly those with Child–Pugh C cirrhosis or Child–Pugh B disease with active bleeding. [6, 20] However, the role of p‐TIPS in gastric variceal bleeding has historically been less well‐defined. Earlier guidance highlighted the limited randomized evidence in fundal or isolated gastric varices, and Baveno VII identified p‐TIPS in gastric varices as a research priority. [4] Our findings, incorporating the recent GAVAPROSEC trial, [13] extend this evidence base by providing more robust randomized data supporting p‐TIPS in gastric variceal bleeding, thereby strengthening its applicability beyond esophageal varices and aligning with the evolving guideline landscape.

The current findings are largely in agreement with previously published meta‐analyses. The individual patient data meta‐analysis by Nicoară‐Farcău et al. (2024) found that pre‐emptive TIPS significantly increased survival in high‐risk patients, with improved control of bleeding and ascites and no increase in the risk of hepatic encephalopathy. [10] Similarly, a network meta‐analysis by Huang et al. (2024) reported reduced all‐cause mortality and rebleeding with early TIPS, without a significant increase in hepatic encephalopathy. [12] The Society of Critical Care Medicine guideline meta‐analysis (Halabi et al.) found decreased 1‐year mortality (RR 0.68) and rebleeding (RR 0.28) with early TIPS, with no significant difference in encephalopathy. [21] The systematic review by Zhou et al. (2021) also reported significant reductions in mortality (RR 0.64) and rebleeding (RR 0.40) with early TIPS, with the greatest improvements in Child–Pugh C and Child–Pugh B with active bleeding. [11] In contrast, the Cochrane review (Simonetti et al., 2020) found very low certainty in the evidence for mortality and rebleeding outcomes, emphasizing the need for higher‐quality trials. [22]

However, the picture is not entirely uniform. Hussain et al. (2022) demonstrated that pooled RCT data did not reach statistical significance for mortality, highlighting the need for larger trials. [23] The AASLD Practice Guidance (2024) notes that a recent RCT did not demonstrate a significant survival benefit, although the risk of rebleeding was lower with pre‐emptive TIPS. [24] Subgroup analysis by Child–Pugh classification demonstrated that the mortality benefit of p‐TIPS was maintained regardless of disease severity, suggesting that the effect extends beyond the most severely decompensated cohort. Importantly, the current meta‐analysis is the first to incorporate the GAVAPROSEC trial, extending the evidence base to patients with fundal gastric variceal bleeding (GOV2 and isolated gastric varices). In this subgroup, p‐TIPS was associated with a marked reduction in the risk of rebleeding, an endpoint that prior meta‐analyses had not quantified. This expands the clinical applicability of pre‐emptive TIPS and supports its use in a population underrepresented in prior studies. [24, 6]

Several features distinguish the current meta‐analysis from prior work. By restricting inclusion to RCTs, the analysis minimizes the selection and confounding bias inherent in observational data. The inclusion of studies from diverse geographical regions (Asia and Europe) further enhances the external validity and generalizability of the findings. Comprehensive subgroup analyses stratified by Child–Pugh class and variceal location further allow for more precise and individualized clinical recommendations. Beyond the conventional endpoints of mortality and rebleeding, this analysis also captures a broader spectrum of outcomes, including ascites, spontaneous bacterial peritonitis, hepatorenal syndrome, and liver‐related death, offering a more complete picture of the systemic impact conferred by portal pressure reduction and surpassing the scope of most previous meta‐analyses. [11, 25, 26] The robust reduction in rebleeding risk observed in patients with fundal gastric varices is a particularly important advance, with meaningful implications for a subgroup that has long lacked dedicated evidence to guide management.

This meta‐analysis has several limitations that warrant consideration. The modest total sample size may reduce statistical power for rare outcomes and limit the robustness of some subgroup analyses. The exclusion of observational data, while improving internal validity, may limit generalizability to real‐world populations. The moderate heterogeneity in our analysis likely reflects variability in patient selection, variceal subtype, and TIPS timing across included trials. A majority of the included RCTs focused on patients with alcohol‐related liver disease, which simultaneously provides methodological homogeneity for this specific etiology while imposing a limitation on the generalizability of the findings to nonalcoholic liver disease populations. [19, 7] Etiology‐specific subgroup data were not consistently reported across the included trials, precluding pooled synthesis by cirrhosis cause. Future randomized studies should prospectively stratify outcomes by etiology to determine whether treatment effects differ between alcohol‐related and nonalcohol‐related liver disease

The severity, prognosis, and response to therapeutic interventions can differ significantly based on the cause of cirrhosis. Patients with alcohol‐related liver disease (ArLD), particularly those with continued alcohol use, often have worse underlying nutritional status and may experience more rapid clinical deterioration than patients with, for example, compensated viral hepatitis or primary biliary cholangitis. [27] An additional limitation is the restriction of this meta‐analysis to clinical endpoints, omitting health‐economic data. Consequently, a definitive statement on the cost‐effectiveness of the p‐TIPS strategy, particularly when compared with repeated endoscopic interventions, cannot be established. Finally, most included studies report outcomes within a 6‐week to 1‐year window, leaving the long‐term effects of p‐TIPS less well characterized. [22, 23, 25]

## 5. Conclusion

This meta‐analysis provides robust RCT‐based evidence that p‐TIPS significantly reduces mortality and rebleeding in patients with AVB without increasing the risk of hepatic encephalopathy. These findings support broader adoption of p‐TIPS in high‐risk patients and reinforce its integration into clinical practice guidelines. Future trials with larger sample sizes and extended follow‐up are warranted to further define optimal patient selection and long‐term outcomes.

## Author Contributions

Wan Muhammad Izhar bin Wan Ismail, Susmitha Chalamalasetti, and Faraz Iqbal contributed equally and are co‐first authors.

## Funding

No funding was received for this manuscript.

## Conflicts of Interest

The authors declare no conflicts of interest.

## Supporting information


**Supporting Information** Additional supporting information can be found online in the Supporting Information section. Figures S1–S12, including the PRISMA study‐selection flow diagram, risk‐of‐bias assessment, subgroup analyses of all‐cause mortality and rebleeding according to variceal bleeding type and Child–Pugh class A composition, and forest plots for new or worsening ascites, hepatorenal syndrome, liver‐related mortality, spontaneous bacterial peritonitis, serious adverse events, and hepatic encephalopathy comparing pre‐emptive TIPS with standard care.

## Data Availability

The data that support the findings of this study are available from the corresponding author upon reasonable request.
