# Supplementary material for: Pre‐Emptive TIPS for Acute Variceal Bleeding: A Systematic Review and Meta‐Analysis of Randomized Controlled Trials
Source: Int J Hepatol. 2026 Sep 2;2026:3244544. doi: 10.1155/ijh/3244544 (PMC13539072; doi:10.1155/ijh/3244544)
Supplement: Supplementary file 1 [file IJH-2026-3244544-s001.docx]

**Supplementary Material**

**"Pre-emptive TIPS for acute variceal bleeding: A systematic review and meta-analysis of RCTs"**

**Supplementary Figure S1.** PRISMA flow diagram illustrating the study selection process.

**Supplementary Figure S2**. Risk of bias summary across five domains

**Supplementary Figure S3.** Subgroup analysis of all-cause mortality by type of variceal bleeding comparing pre-emptive TIPS versus standard care.

**Supplementary Figure S4.** Subgroup analysis of all-cause mortality by Child-Pugh class A composition of included trials comparing pre-emptive TIPS versus standard care.

**Supplementary Figure S5.** Subgroup analysis of rebleeding by type of variceal bleeding comparing pre-emptive TIPS versus standard care.

**Supplementary Figure S6.** Subgroup analysis of rebleeding by Child-Pugh class A composition of included trials comparing pre-emptive TIPS versus standard care.

**Supplementary Figure S7.** Forest plot of new or worsening ascites comparing pre-emptive TIPS versus standard care.

**Supplementary Figure S8.** Forest plot of hepatorenal syndrome comparing pre-emptive TIPS versus standard care.

**Supplementary Figure S9.** Forest plot of liver-related mortality comparing pre-emptive TIPS versus standard care.

**Supplementary Figure S10.** Forest plot of spontaneous bacterial peritonitis comparing pre-emptive TIPS versus standard care.

**Supplementary Figure S11.** Forest plot of serious adverse events comparing pre-emptive TIPS versus standard care.

**Supplementary Figure S12.** Forest plot of hepatic encephalopathy comparing pre-emptive TIPS versus standard care.
